# Measuring Antimicrobial Use Needs Global Harmonization

**DOI:** 10.1002/gch2.202100017

**Published:** 2021-06-10

**Authors:** Muhammad Umair, Mashkoor Mohsin, Ute Wolff Sönksen, Timothy Rutland Walsh, Lothar Kreienbrock, Ramanan Laxminarayan

**Affiliations:** ^1^ Institute of Microbiology University of Agriculture Faisalabad 38000 Pakistan; ^2^ Statens Serum Institut Copenhagen 2300 Denmark; ^3^ Department of Zoology INEOS Oxford Institute for AMR Research University of Oxford Oxford OX1 3SZ UK; ^4^ Department for Biometry Epidemiology and Information Processing University of Veterinary Medicine Hannover 30559 Hannover Germany; ^5^ Center for Disease Dynamics Economics and Policy Washington DC 20005 USA

**Keywords:** AMU, global harmonization, metrics

## Abstract

Global health and global economies are predicted to be severely affected by antimicrobial resistance (AMR). The three organizations World Health Organization/World Organisation for Animal Health/Food and Agriculture Organization (WHO/OIE/FAO) are working in their domains to prevent any future AMR crisis. Antimicrobial use (AMU), especially in food animals, is contributing to the development and dissemination of AMR bacteria and genes. AMU monitoring is a strategic objective of the global and national action plans on AMR. However, the AMU reporting metrics at different levels are not harmonized yet, posing difficulties in comparisons among AMU data from different sources. A tripartite WHO/OIE/FAO collaboration is urgently required to develop and implement a globally accepted AMU metric system to ensure reliable comparisons among various data sets.

## Graphical abstract

Antimicrobial resistance action plans by World Health Organization (WHO), World Organisation for Animal Health (OIE), and Food and Agriculture Organization (FAO) call for the monitoring and surveillance of antimicrobial use (AMU) in human and animal health sectors. However, AMU reporting methodologies and metrics differ among various systems and countries. A tripartite WHO/OIE/FAO collaboration is required to develop and implement a globally acceptable AMU metric system.

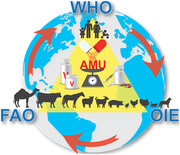

## Perspective

Antimicrobial resistance (AMR) is an emerging threat to global health and economy.^[^ ^1^ ^]^ To continue the fight against this major transboundary crisis, the World Health Organization (WHO) adopted a global action plan on AMR (GAP‐AMR) in the year 2015.^[^ ^2^ ^]^ The World Organisation for Animal Health (OIE) and the Food and Agriculture Organization (FAO) also joined the WHO to mitigate AMR crisis. The tripartite organizations (WHO, OIE, and FAO) are working to assist countries in preparing and implementing their national action plans on AMR (NAPs‐AMR). In agreement with GAP‐AMR, more than 115 countries had adopted their NAPs‐AMR by the year 2019.^[^ ^3^ ^]^

Excessive antimicrobial use (AMU) in food animals has been associated with the development and dissemination of AMR bacteria and genes. Monitoring AMU in humans and animals is an essential strategic objective of GAP‐ and NAPs‐AMR. Therefore the tripartite organizations, the UK Fleming Fund, the Bill and Melinda Gates Foundation, and others are already strengthening a number of developing countries’ resources and capacities to monitor AMU. However, many countries still lack sufficient fundamental resources to sustainably support medical or veterinary services and facilities enabling them to produce and publish accurate regional/national or hospital/farm‐level AMU data. To quantify the national‐ or hospital‐level human AMU data, WHO has standardized an anatomical therapeutic chemical/defined daily dose (ATC/DDD) system.^[^ ^4^ ^]^ The WHO report on surveillance of antibiotic consumption includes estimates from 65 countries and areas based on data from different sources, i.e., imports, sales, prescriptions, etc.^[^ ^5^ ^]^ Concordantly, in the animal health sector, OIE introduced a template to collect national‐level animals AMU data based on amounts in milligrams of active ingredient used over adjusted animal biomass. In the fifth OIE annual report on antimicrobial agents intended for use in animals, published in 2021, 133 of 182 member countries have submitted their quantitative AMU data.^[^ ^6^ ^]^ However, the heterogeneity of livestock systems as well as the heterogeneity of administrative processes within the countries participating is narrowing the veracity of these data sets for direct comparisons.

OIE is standardizing the national‐level AMU reporting methodology, however, it differs from the national‐level AMU reporting methodologies previously proposed by WHO and European Medicine Agency (EMA) (**Table** **1**).^[5–7]^ This is partly due to the wish to accommodate different countries with differing health and production systems. ATC/DDD system defined by WHO is based on recommended daily dose of antimicrobial for an average adult body weight of 70 kg.^[^ ^4^ ^]^ However, in case of animals there are different species that could be potentially treated with antimicrobials in each country, their average weights are still required to be standardized nationally/regionally/globally.^[^ ^7^ ^]^ Moreover, different countries are using various methodologies/indicators to quantify their national‐level AMU data in human and animal health sector.^[^ ^5^, ^6^ ^]^

**Table 1:** Different indicators to quantify antimicrobial consumption and use as defined by various agencies

Agencies	AMU indicators		Numerators	Denominators
	National level—AMC data	Hospital/Farm level—AMU data		
WHO	$\frac{\text{DDDs}\;\text{used}}{1000\;\mathrm{inhibitant}-\text{days}}$ UD defined for combination products^[^ ^5^ ^]^	Number of antibiotics prescribed per patient, ATC/DDD system^[^ ^4^, ^13^ ^]^	DDD	Population size
OIE	$\frac{\text{Antimicrobial}\;\text{agents}\;\text{reported}\;(\text{mg})}{\text{Animal}\;\text{biomass}\;(\text{kg})}$ Data reported from sales, purchase, imports, prescriptions, farm records (use data)^[^ ^6^ ^]^	–	AAI	Animal biomass
EMA	$\frac{\text{Amount}\;\text{sold}\;(\text{tonnes})}{\text{PCU}}$ PCU is a most likely theoretical animal weight in kg at the time of treatment.^[^ ^7^ ^]^	i. $\frac{\text{Active}\;\mathrm{substance}\;(\text{mg})}{\text{PCU}}$ ii. $\frac{\text{DDDvet}}{\text{PCU}}$ iii. $\frac{\text{DCDvet}}{\text{PCU}}$ ^[^ ^12^ ^]^	AAI, DDDvet, DCDvet	PCU
AACTING	–	$\frac{\text{DDDs}\;\text{used}}{100\;\text{animal}-\text{days}}$ ^[^ ^11^ ^]^	DDDs	Population size

AACTING: network on quantification of veterinary antimicrobial usage at herd level and analysis, communication, and benchmarking to improve responsible usage; AAI: antimicrobial active ingredient; AMC: antimicrobial consumption; AMU: antimicrobial use; ATC: anatomical therapeutic chemical; DDDs: defined daily doses; DDDvet: defined daily doses for animals; DCDvet: defined course doses for animals; EMA: European Medicine Agency; OIE: World Organisation for Animal Health; PCU: population correction unit; UD: unit dose; WHO: World Health Organization.

Data on high‐resolution AMU surveillance (AMU surveillance at hospital, farm, or species level) is scarce particularly from low‐ and middle‐income countries (LMICs). Additionally, high‐resolution AMU surveillance methodology has not yet been successfully harmonized as different studies are reporting AMU using varying metrics and abbreviations which poses difficulties in comparing AMU data from different sources leading to unreliable interpretation.^[^ ^8^, ^9^, ^10^ ^]^ EMA and AACTING proposed guidelines to quantify antimicrobial use at farm/species level; however, the proposed indicators are unmatched (Table 1).^[^ ^11^, ^12^ ^]^

**Figure 1:**
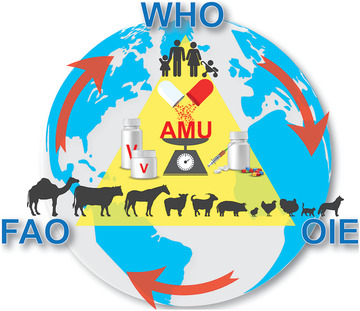
WHO/OIE/FAO tripartite collaboration on harmonization of antimicrobial use metrics.

To achieve uniformity among AMU data collection across the countries and to ensure reliable AMU comparisons among different data sets including humans and animals, a tripartite WHO/OIE/FAO collaboration is required to develop and implement a globally acceptable AMU metric and abbreviation system built on existing initiatives by WHO, OIE, EMA, AACTING, etc. **Figure** **1**. The system should clearly describe and define which metrics are recommended for different monitoring purposes—from stabile, sustainable surveillance of trends over time, to comparison between different species and production systems to benchmarking. Consequently, this will facilitate evaluating antimicrobial stewardship measures in human and animal health sector.

## Conflict of Interest

The authors declare no conflict of interest.
